# Plasma and Kidney Proteome Profiling Combined with Laser Capture Microdissection Reveal Large Increases in Immunoglobulins with Age

**DOI:** 10.3390/proteomes12020016

**Published:** 2024-06-03

**Authors:** Leanne J. G. Chan, Niclas Olsson, Magdalena Preciado López, Kayley Hake, Haruna Tomono, Matthew A. Veras, Fiona E. McAllister

**Affiliations:** Calico Life Sciences LLC, 1130 Veterans Blvd, South San Francisco, CA 94080, USA

**Keywords:** proteomics, laser capture microdissection, aging, kidney, immunoglobulins, complement, plasma

## Abstract

One of the main hallmarks of aging is aging-associated inflammation, also known as inflammaging. In this study, by comparing plasma and kidney proteome profiling of young and old mice using LC–MS profiling, we discovered that immunoglobulins are the proteins that exhibit the highest increase with age. This observation seems to have been disregarded because conventional proteome profiling experiments typically overlook the expression of high-abundance proteins or employ depletion methods to remove them before LC–MS analysis. We show that proteome profiling of immunoglobulins will likely be a useful biomarker of aging. Spatial profiling using immunofluorescence staining of kidney sections indicates that the main increases in immunoglobulins with age are localized in the glomeruli of the kidney. Using laser capture microdissection coupled with LC–MS, we show an increase in multiple immune-related proteins in glomeruli from aged mice. Increased deposition of immunoglobulins, immune complexes, and complement proteins in the kidney glomeruli may be a factor leading to reduced filtering capacity of the kidney with age. Therapeutic strategies to reduce the deposition of immunoglobulins in the kidney may be an attractive strategy for healthy aging.

## 1. Introduction

Inflammation and inflammaging are often associated with an increase in the abundance of inflammatory markers such as cytokines. However, immunoglobulins, the main effector molecules of the immune system, surprisingly have not been profiled in detail in the context of mouse aging. Many of the plasma aging studies to date have been performed using assay-based technology that relies on either antibodies (e.g., Olink’s Proximity Extension Assay technology) or aptamers (e.g., SomaScan from SomaLogic). These assays do not normally include the ability to analyze high-abundance plasma proteins such as immunoglobulins; therefore, high-abundance protein profiling data is currently lacking for these methods. The other main method for protein profiling is liquid chromatography–mass spectrometry (LC–MS) proteomics. However, for plasma LC–MS profiling, samples are often depleted of high-abundance proteins (e.g., albumin, immunoglobulins, and complement) prior to analysis to increase the number of low-abundance proteins quantified due to the dynamic range problem of plasma proteins [1].

To gain an unbiased view of the changes in proteins in plasma with age, we profiled young and old mouse plasma samples. By using undepleted plasma samples, we aimed to gain insights into medium- to high-abundance proteins in an unbiased fashion using LC–MS proteome profiling. 

Mice and humans have five major immunoglobulin isotypes: IgG, IgA, IgM, IgD, and IgE. To our knowledge, no in-depth, unbiased mass spectrometry-based proteome profiling has previously been performed on undepleted mouse plasma with age. However, there have been a number of studies profiling Igs in human plasma, and there are contradictions in the literature as to whether immunoglobulins increase or decrease with age. The reasons behind these contradictory reports are likely a result of the different age ranges being compared, different isotypes, as well as the different methodologies employed. The largest human dataset published to date for Ig profiling is from the Rotterdam study, in which they measured over 8000 serum samples using a turbidimetric immunoassay for IgG, IgA, and IgM [2]. In this study, they observed an increase in IgG after age 65 but a decrease from age 45–65, an increase in IgA, and no significant changes in IgM with age. In another human study involving 2000 patients in which Ig levels were measured with nephelomy, it was observed that IgG and IgM concentrations were reduced in old age and that men (but not women) showed an increase in IgA with age [3]. A study that profiled Igs using nephelometric techniques in 166 subjects (20–106 years) reported an increase in IgG and IgA with age but decreases in IgD and IgM [4]. Another study [5] that measured 86 human plasma samples from age 22 to 111 using mass spectrometry proteomics quantified 410 proteins. This study observed that various IgG proteins (heavy and light chains) and IgM were decreased with age. It is worth noting that in another large study [6] from the Wyss-Coray group that used the SomaScan assay, the only Ig that was detected was an aptamer reagent that was described as binding to ‘IGHE.IGK.IGL’ and so presumably targeted the IgE isotype and was observed not to change with age. No data for IgG, IgA, or IgM were included in that study given the limitations of the technology covering only the lower abundant proteins.

Although there is no work published to date that profiles the proteome of young and old mouse plasma in an unbiased, discovery fashion, there have been multiple studies that have profiled young and old mouse tissues using discovery proteomics. The largest study to date [7] profiled 10 tissues from 20 mice and observed that immune proteins, and in particular immunoglobulins, were the proteins that increased most with age across almost all tissues. This is also consistent with an earlier paper [8] from The Tabula Muris Senis consortium that observed widespread immune cell activation and increases in immunoglobulin genes in multiple mouse organs with age. In this landmark paper, IgJ increases were linked to the infiltration of plasma cells into multiple tissues and, in particular, in adipose tissue. Interestingly, a very recent publication in *Cell Metabolism* from the Qiang research group showed that IgG is an aging factor that drives adipose tissue fibrosis and metabolic decline. The data from this paper suggests that IgG could be useful not just as a biomarker of aging but also as a therapeutic target.

The contradictory reports of Ig changes with age in plasma in the literature likely arise from the precise age ranges compared, the precise method of Ig quantification, as well as the exact isotypes that are quantified. In addition, the mouse IgG isotype is subdivided into IgG1, IgG2a, IgG2b, IgG2c, and IgG3, each of which has slightly different functions. Immunoglobulins play a crucial role in the immune system for both the innate and adaptive immune response. Immunoglobulins have both an Fc (fragment crystallizable) portion, also known as the constant region, and an antigen-binding domain. Immunoglobulins have a number of roles, including the recognition of pathogens (such as bacteria, viruses, fungi, and foreign substances), the opsonization and neutralization of pathogens, the activation of the complement system and immune cells, as well as the longer-term memory response. The antigen-binding domain of immunoglobulins is highly diverse due to somatic hypermutation and class switching. The Fc region in immunoglobulins is responsible for interaction with effector cells through FcRs (Fc receptors) on various immune cells, such as macrophages, neutrophils, and natural killer and mast cells.

The kidney is the organ responsible for filtering plasma proteins and the organ most sensitive to changes in plasma protein concentrations. Decreased kidney filtering capacity is known to occur with both age and disease. In addition, consistent structural changes are known to occur in the kidney with age, such as the loss of nephrons and sclerosis. However, the precise mechanisms behind the drivers of sclerosis and loss of filtration are not known. Since immunoglobulins are high-abundance plasma proteins and the kidneys are responsible for filtering plasma proteins, we sought to determine if there is any connection between aging kidneys and serum immunoglobulins by profiling young and old kidneys.

## 2. Materials and Methods

### 2.1. Materials

Lysyl endopeptidase (Lys-C) was obtained from FUJIFILM Wako Chemicals (Richmond, VA, USA). Sequencing Grade Modified Trypsin and Trypsin/Lys-C Mix were obtained from Promega Corporation (Madison, WI, USA). cOmplete ULTRA protease inhibitor tablets and PhosSTOP phosphatase inhibitor tablets were obtained from Roche (Mannheim, Germany). HEPES and EPPS buffers were obtained from Boston BioProducts (Milford, MA, USA). T-PER, Halt Protease Inhibitor Cocktail, DynaMag-2 Magnet, Pierce BCA Protein Assay Kits, Pierce FlexMix Calibration Solution, TMT 10-plex Label Reagent Set, and the TMTpro 18-plex Label Reagent Set were obtained from Thermo Fisher Scientific (Waltham, MA, USA). Sep-Pak C18 solid-phase cartridges were obtained from Waters Corporation (Milford, MA, USA), and Protein LoBind tubes were from Eppendorf (Enfield, CT, USA). All other reagents were obtained from Sigma-Aldrich (St. Louis, MO, USA) unless specified otherwise.

### 2.2. Mice

Young and old C57BL/6 mice were purchased from The Jackson Laboratory (4 months and 32 months for the 10-plex experiment) or Charles River Laboratory (3 months and 21 months for the 18-plex experiment). The mice were housed at Calico for at least one week prior to the start of studies in order to acclimate them to the facilities. The mice were maintained at a constant temperature (22 ± 1 °C) and humidity (60–70%), and all experiments were performed during the day phase of a 12/12 light–dark (LD) cycle. Calico is committed to conducting ethical animal experimentation and the 3Rs principle (Replacement, Reduction, and Refinement). The Institutional Animal Care and Use Committee (IACUC) at Calico approved the animal studies. Studies were performed in an AAALAC-accredited facility, with veterinary supervision ensuring adherence to the highest standards of animal care and oversight.

All mice were sedated by the inhalation of isoflurane (1.5–2.5%, 0.8–1.0 L/min oxygen flow rate). Blood was collected in EDTA by cardiac puncture, and then plasma was isolated and stored at −80 °C until needed for analyses. Mice were perfused with ice-cold PBS for 5 min, and then the kidneys were harvested and either flash-frozen in liquid nitrogen or embedded in OCT blocks. Kidney tissues were stored at −80 °C until ready for analysis.

### 2.3. LC–MS Sample Preparation for Mouse Plasma and Kidneys

Plasma (20 µL) was diluted to 150 µL (8 M urea/50 mM HEPES pH 8.5). Frozen kidney samples were homogenized in lysis buffer (75 mM NaCl, 50 mM HEPES pH 8.5, 3% SDS, with protease (cOmplete ULTRA) and phosphatase (PhosSTOP) inhibitor tablets then centrifuged for 5 min at 16,000× *g* to pellet cellular debris. Proteins were reduced with dithiothreitol (DTT, 5 mM, 37 °C for 30 min) and then alkylated with iodoacetamide (15 mM, room temperature for 20 min in the dark). DTT was used to quench excess iodoacetamide (5 mM, room temperature for 15 min in the dark).

Proteins from kidney samples (400 μg) were extracted by a methanol–chloroform precipitation method in which methanol, chloroform, and water were sequentially added (4:1:3:1 methanol–chloroform–water–sample) to the samples, with vortexing after each reagent addition. Phase separation was induced by centrifugation (30 min, 16,000× *g* at 4 °C), and then the top and bottom liquid layers were removed to isolate the protein pellets. The protein pellets were washed with 3× sample volumes of methanol and then centrifuged for 10 min (16,000× *g* at 4 °C). All remaining liquid was removed then protein pellets were dried (RT, 10 min). The dried protein pellets were resuspended in 8 M urea/50 mM HEPES, pH 8.5.

The samples were diluted to 4 M urea/50 mM HEPES, pH 8.5, and then digested with Lys-C (25 °C, overnight). The samples were further diluted (to 1 M urea/50 mM HEPES pH 8.5) and then digested with trypsin (5 ng/µL, 6 h at 37 °C). The digests were quenched and acidified with 0.5% trifluoroacetic acid (TFA) and then desalted (Sep-Pak C18 cartridges). The samples were eluted from the Sep-Pak columns using 40% acetonitrile/0.5% acetic acid followed by 80% acetonitrile/0.5% acetic acid and then dried completely (SpeedVac vacuum concentrator). Peptide concentrations were quantified (BCA Assay Kit, Pierce), and then each sample was aliquoted (100 µg) and dried (SpeedVac).

The peptide aliquots were resuspended in 100 µL (200 mM HEPES, pH 8.5/20% acetonitrile) and labeled with tandem mass tag (TMT). Next, 400 µg of TMT was added to label each sample. The samples were incubated for 1 h at 25 °C with shaking, and then excess TMT was quenched with hydroxylamine (final concentration 1%). The samples were pooled at equal peptide ratios into a 10-plex or 18-plex, and then the combined sample was desalted using a Sep-Pak C18 cartridge and dried in the SpeedVac.

The sample was resuspended in Buffer A (5% acetonitrile, 10 mM ammonium bicarbonate) and then fractionated using high-pH reversed-phase chromatography (Agilent 1260 UPLC with a diode array detector utilizing 3 wavelengths—215, 220, and 254 nm). Peptides were loaded onto a ZORBAX Extend-C18 column (Agilent, 250 mm length × 4.6 mm ID, 5 µm particle size) operating at 25 °C with a 0.5 mL/min flow rate. The column was equilibrated to starting conditions of 100% Buffer A and 0% buffer B (95% acetonitrile, 10 mM ammonium bicarbonate), and then the peptides were eluted by the following gradient: B was increased to 35% B over 60 min, then increased to 100% B over 5 min and held at 100% B for 5 min. The samples were separated into 96 fractions and then pooled into 24 fractions, as previously described [9]. The pooled fractions were dried completely (SpeedVac) and then resuspended for LC–MS analysis (5% ACN/5% formic acid).

### 2.4. Multiplexed Immunoassay to Quantify Antibody Isotypes in Plasma

Antibody isotypes in the plasma (8 young mice—4 months, 7 old mice—32 months) were quantified using a 7-plex ProcartaPlex Mouse Panel (Thermo Fisher Scientific, EPX070-20815-901) according to the manufacturer’s protocol. Plasma samples were diluted between 5000 and 20,000 fold. A FLEXMAP 3D System equipped with xPONENT Software version 4.3 (Luminex, Austin, TX, USA) was used to run all assays. Assay settings for all runs were based on the manufacturer’s instructions, except high PMT was used for the reporter gain. ProcartaPlex Analysis software version 3.1.0 (Thermo Fisher Scientific) was used to analyze all data. 

### 2.5. Immunofluorescence of Kidney Tissue Sections

Fresh-frozen OCT-embedded kidney tissues were sectioned to a thickness of 15 µm and mounted on glass slides, then fixed in 4% paraformaldehyde (Electron Microscopy Sciences, Hatfield, PA, USA, 15710) in PBS (20 min, room temperature). Samples were permeabilized in 0.1% Triton™ X-100 (Sigma, T8787) in PBS (10 min, room temperature). Samples were blocked in 5% BSA (Sigma, A2153) in PBS-T (PBS with 0.001% Tween 20, Sigma, P9416) to reduce non-specific binding (1 h, room temperature), then incubated with primary antibody (5 µg/mL, Table 1) in blocking buffer (overnight, 4 °C). Samples were washed with PBS-T (3 times, room temperature), then incubated with secondary antibodies (1:500 dilution, Table 1) in blocking solution (1 h, room temperature) as needed. Samples were washed thoroughly in PBS-T (3–5 times over 2 h, room temperature), then washed in PBS with 1:100 dilution of Alexa Fluor 647 Phalloidin (ThermoFisher, A22287) (10 min, room temperature). The slides were gently dried using a Kimwipe and then mounted using ProLong Gold Antifade Mountant with DAPI (ThermoFisher, P36931) with a square cover glass (Corning, 2850-22; Corning, NY, USA). Fluorescence images were captured with a Nikon Eclipse Ti equipped with a CSU-X1 Confocal Scanner Unit (Yokogawa; Tokyo, Japan), four laser lines (405, 488, 561, and 640 nm), and an Andor iXon Ultra EMCCD camera. NIS-Elements AR 5.21.03 software was used to produce multi-color single-plane confocal images, and the images were analyzed with ImageJ/FIJI version 2.14.0 [10].

### 2.6. Preparation of Slides for Laser Capture Microdissection

Polyethylene terephthalate (PET) membrane slides with metal frames (Leica, 11505190; Wetzlar, Germany) were treated with UV light for 30 min, and then fresh-frozen OCT-embedded kidney tissue sections (15 μm thick) were mounted onto the membranes. Laser capture microdissection (LCM) slides were stored at −80 °C until ready for microdissection. To prepare for LCM, the slides were thawed at −20 °C for 15 min followed by 4 °C for 15 min, then placed on a cold thermal tray prechilled on wet ice (Corning, CLS432074). The slides were washed 3 times with PBS to remove the OCT, then washed 3 times with 20 mM HEPES, pH 7.5, and drained with a Kimwipe. A second PET membrane slide was placed directly on top of the washed LCM slide to make a sandwich, and the extra liquid was carefully removed by scraping the membrane with a plastic razor (VWR, 10048-876; Radnor, PA, USA). 

### 2.7. Laser Capture Microdissection

Slide sandwiches were passed through an antistatic device (VWR, 11238-356) to help with tissue detachment after cutting. Glomeruli and tubules were identified using phase contrast, and shapes for laser capture were drawn manually to capture approximately 0.5 mm^2^ of each (~50 glomeruli, ~75 tubules). Laser microdissection was performed with the LMD7 (Leica) using the “Draw + Cut” method with “Laser Screw”, in which the laser passed 2–3 times around the shape with the following settings: power 47, aperture 13, speed 7, head current 100%, pulse frequency 1019, and specimen balance 19. Using gravity, glomeruli and tubules were sorted and collected in separate 0.6 mL Axygen tubes (Corning, MCT-060-L-C) containing 25 µL of T-PER with 1X Halt protease inhibitor. The microdissected tissue samples were stored at −80 °C until preparation for LC–MS analysis.

### 2.8. LCM Sample Preparation for Spatial Proteome Profiling

Frozen glomerulus and tubule samples were thawed on ice and then sonicated for 5 min in an ice bath to homogenize the microdissected tissues. Proteins were reduced with DTT (5 mM, 30 min, 25 °C, 800 rpm) and alkylated with iodoacetamide (15 mM, 30 min, 25 °C in the dark, 800 rpm), and then the reaction was quenched with DTT (5 mM, 15 min, 25 °C in the dark, 800 rpm).

Protein cleanup was performed using a 1:1 mixture of E3:E7 Sera-Mag Carboxylate-Modified Magnetic Particles (Cytiva Life Sciences, 44152105050350, 24152105050350; Marlborough, MA, USA). The beads were washed with water 3 times, and then the sample was added onto the beads. For protein binding, acetonitrile was added to a concentration of 75%, and then the mixture was incubated for 20 min at room temperature without shaking. The beads were immobilized on a magnet and then washed twice with 200 µL of 70% ethanol, followed by 2 washes with 200 µL of 100% acetonitrile. The beads were resuspended in 46 µL of digestion buffer (50 mM EPPS, pH 8.5, 10 mM CaCl_2_), and then 1 µg of Trypsin/Lys-C Mix (Promega, V5071) was added, followed by incubation for 1 h at 37 °C (1000 rpm) to digest the proteins.

The digested samples were immobilized on a magnetic rack, and the supernatants (peptides) were transferred to a new tube. Isobaric labeling was performed by adding 0.2 mg of TMTpro reagent to each sample and incubating for 1 h at 25 °C (600 rpm). Excess TMT was quenched by adding hydroxylamine to a final concentration of 0.5% (*v*/*v*). The samples were combined into a single plex and then cleaned using StageTip as previously described [11]. The eluted StageTip sample was dried to completion in a SpeedVac vacuum concentrator and resuspended in 5% acetonitrile/5% formic acid for LC–MS analysis.

### 2.9. TMT Data Acquisition Mass Spectrometry Methods on Orbitrap Fusion Lumos

Samples from plasma (TMT 10-plex and 18-plex) and kidney (TMT 18-plex) were analyzed on an Orbitrap Fusion Lumos Tribrid mass spectrometer using data-dependent acquisition mode. The mass spectrometer was coupled to an EASY-nLC 1200 (Thermo Fisher Scientific). Peptides were separated on an Aurora Series emitter column (25 cm × 75 µm i.d., 1.6 µm, 120 Å pore size, C18; IonOpticks, AUR2-25075C18A), operating at 60 °C and 300 nL/min. The gradient was 165 min with acetonitrile increasing from 8 to 30% in 0.125% formic acid. The mass spectrometer acquisition parameters are detailed below. A high-resolution MS1 scan was collected in the Orbitrap (*m*/*z* range 500–1200, 60 k resolution, AGC 5 × 10^5^, 100 ms max. injection time, RF lens 30%). The maximum cycle time was set to 5 s, and the top 10 precursors were selected for MS2, followed by synchronous precursor selection MS3. For MS2, ions were isolated (0.5 *m*/*z* window) and analyzed in the quadrupole ion trap with the following parameters: collision-induced dissociation (CID), 35% normalized collision energy, 35 ms max. injection time, and AGC 1 × 10^4^. MS3 was analyzed using the Orbitrap with the following parameters: higher energy collision-induced dissociation (HCD), 45% normalized collision energy, 250 ms max. injection time, 60 k resolution, and AGC 5 × 10^4^. A maximum of six fragment ions from each MS2 spectra were selected for analysis by synchronous precursor selection MS3.

### 2.10. TMT Data Acquisition Mass Spectrometry Methods on Orbitrap Eclipse

Data from LCM and 18-plex kidney data were obtained on an Orbitrap Eclipse mass spectrometer. FAIMS (Field Asymmetric Ion Mobility Spectrometry) Pro Interface was used for data collection. The mass spectrometer was coupled to an UltiMate 3000 HPLC operating in DDA mode (Thermo Fisher Scientific). Peptides were separated on an Aurora Series emitter column (25 cm × 75 µm i.d., 1.6 µm, 120 Å pore size, C18; IonOpticks, AUR2-25075C18A) using a 165 min gradient from 8 to 30% acetonitrile in 0.125% formic acid. Real-time search (RTS) with a UniProt mouse database was used for data collection. A high-resolution MS1 scan was performed in the Orbitrap with the following parameters: 120 k resolution, *m*/*z* range 400–1600, RF lens 30%, standard AGC target, and “Auto” max. injection time. The top 10 precursors were selected for MS2. Depending on the RTS, the ions were then analyzed with SPS MS3. For the RTS, the minimum Xcorr needed to pass was set to 1, the minimum dCn was set to 0.1, and the maximum missed cleavages allowed was set to two. The parameters for MS2 acquisition were as follows: 0.7 *m*/*z* isolation window, CID, 35% normalized collision energy, AGC 1 × 10^4^, 35 ms max. injection time) MS3 analysis was performed in the Orbitrap with the following parameters: HCD, 50 k resolution, 45% normalized collision energy, AGC 1 × 10^5^, *m*/*z* range 100–500 *m*/*z*, and 200 ms max. injection time. A maximum of 10 fragment ions from each MS2 spectrum were selected for MS3 analysis using SPS; 10 ppm was set as the mass tolerance for the target masses. Each experiment had a cycle time of 1.25 s.

### 2.11. Analysis of TMT Multiplexed Data

An in-house software pipeline (version 3.12) was used to process all mass spectrometry data [12]. Raw files were first converted to mzXML files. Using the Sequest algorithm, files were searched against a mouse UniProt database (SwissProt database downloaded 18 November 2021) in forward and reverse orientations. The following parameters were used: tryptic peptides, 20 ppm precursor ion tolerance, 0.6 Da product ion tolerance, static carbamidomethylation modifications of cysteine residues (+57.02 Da), static TMT modifications on peptide N-termini and lysine residues (+229.16 Da for 10-plex and +304.20 Da for 18-plex), and differential modification of methionine oxidation (+15.99 Da). Following database searching, linear discriminant analysis (LDA) was performed to filter peptide spectral matches to a 1% false discovery rate (FDR) [12]. Following peptide filtering, non-unique peptides were assigned to proteins that comprised the largest number of matched redundant peptide sequences using the principle of Occam’s razor [12]. The quantification of TMT reporter ions was performed by extracting the most intense ion at the predicted *m*/*z* value for each reporter ion (within a 0.003 *m*/*z* window). Peptide intensities and signal-to-noise ratios were exported and subsequently analyzed using the msTrawler v1 statistical software package [13]. For 10-plex and 18-plex plasma and kidney experiments, a two-condition experimental design (young and old) was used. The LCM data were analyzed primarily with a four-condition experimental design (young tubules, young glomeruli, old tubules, and old glomeruli), and a two-condition analysis was run separately to analyze glomeruli and tubules. Protein fold changes were exported from msTrawler, and gene set enrichment analysis was performed using Advaita Bio’s iPathwayGuide (fold change > 2, *p*-value < 0.05) [14,15].

## 3. Results

### 3.1. Immunoglobulins Increased in Mouse Blood with Age

**Figure 1:**
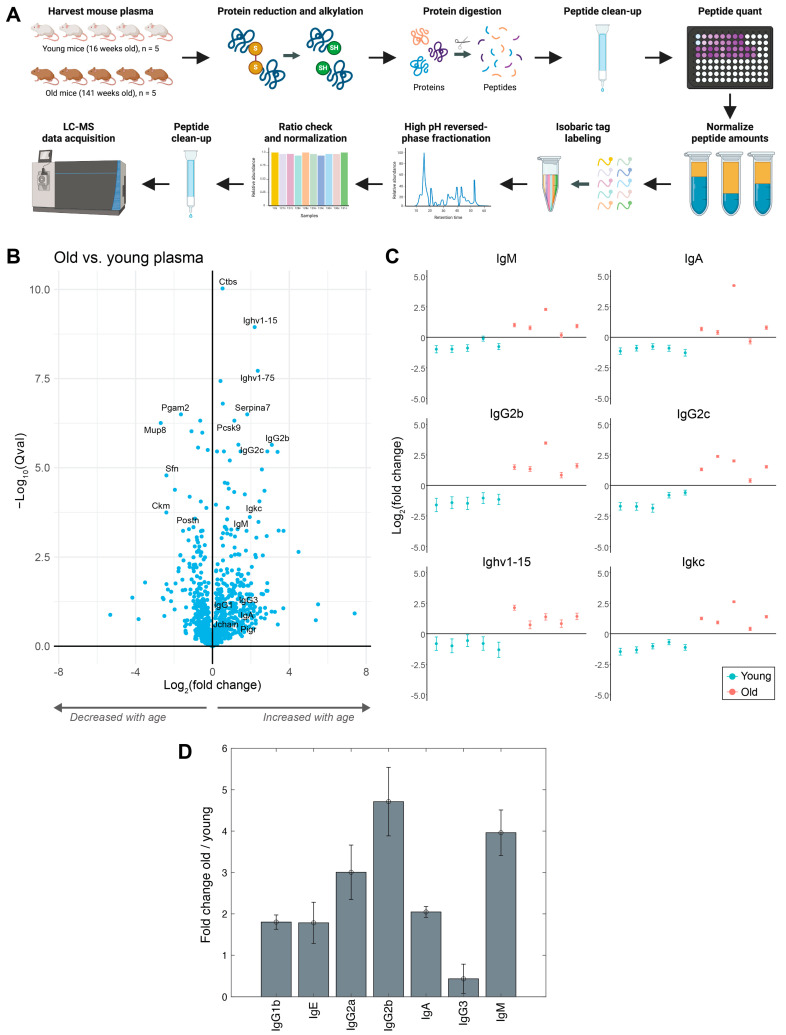
Increase in immunoglobulins with age in mouse plasma. (**A**) Experimental design for multiplexed plasma proteome profiling of young and old mice. (**B**) Volcano plot showing changes between old versus young plasma proteome for 1044 identified proteins (212 proteins with an absolute fold change of >2, 120 proteins with a *q*-value of <0.01). (**C**) Scatter plots of immunoglobulins increased in old mouse plasma proteomics. (**D**) Bar chart showing immunoglobulin fold changes between old vs. young mice from Luminex multiplexed immunoassay. (**A**) was created with Biorender.com (accessed on 30 January 2024).

We first profiled the plasma proteome of undepleted, neat plasma from 10 mice using multiplexed TMT proteomics (Figure 1A). The proteins that increased the most with age included many immunoglobulins (Igs), including IgM, IgA, and IgGs (Figure 1B,C and Figure S6A, Table S1). In addition, Ctbs (di-N-acetylchitobiase), Pcsk9 (proprotein convertase subtilisin/kexin type 9), and Serpina7 (serine or cysteine peptidase inhibitor) significantly increased, while Ckm (creatine kinase M-type), Pgam2 (phosphoglycerate mutase 2), Mup8 (major urinary protein 8), Sfn (14-3-3 protein sigma), and Postn (periostin) significantly decreased with age. We performed gene set enrichment analysis, and the biological processes most enriched with age were immune-related (e.g., immune response, B-cell receptor signaling pathway, complement activation, phagocytosis) (Table S5a). The molecular functions most enriched with age included immunoglobulin receptor binding, antigen binding, and signaling receptor binding (Table S5b). The cellular components most enriched with age included the extracellular region as well as the immunoglobulin complex (Table S5c). To provide further insights into the extracellular region, we extracted all ECM and ECM-associated protein identifications using the Matrisome database [16]. While over 200 ECM proteins were identified, only 15 changed significantly with age in the plasma (fold change > 2) (Figure S7A).

We profiled the same plasma samples using a multiplexed immunoassay (Luminex) and observed similar large increases in most Ig isotypes (Figure 1D). The immunoassay Ig isotype data validated the mass spectrometry proteomics data, with increases observed across all Ig isotypes with the exception of IgG3. To verify whether this observation was consistent in a separate cohort of mice, an additional 18 mice were subsequently profiled (9 young—3 months, 9 old—21 months) (Figures S1A and S5A). Similar to the first cohort, Igs were among the proteins that increased most significantly with age (Figure S1B,C, Table S3). 

### 3.2. Immunoglobulins Increased in Mouse Kidney with Age

**Figure 2:**
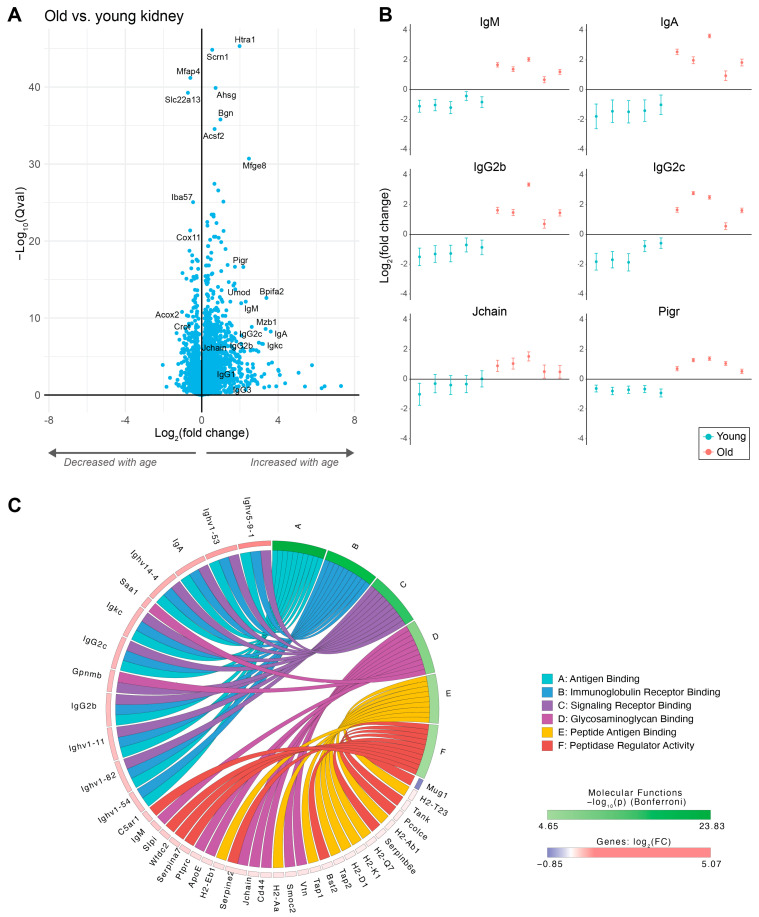
Increase in immunoglobulins with age in the mouse kidney. (**A**) Volcano plot showing changes between old versus young kidneys for 8684 identified proteins (315 proteins with an absolute fold change of >2, 2863 proteins with a *q*-value of <0.01). (**B**) Scatter plots of proteins increased in old kidneys. (**C**) Chord diagram from pathway analysis showing the top six molecular functions enriched with age. Figure obtained using iPathway Guide (Advaita Bio).

The kidney is known to be sensitive to protein deposition as a result of the high blood flow relative to the mass of the kidney, its anatomic structure, and its unique role in filtering blood and concentrating urine. The kidney has previously been shown to be predisposed to the deposition of complement and immunoglobulin proteins with disease [17]. We hypothesized that the large increase in immunoglobulins in the plasma with age may result in an increased deposition of immunoglobulins in the kidney with age. We profiled the kidney proteome of 10 mice (5 young—4 months, 5 old—32 months) using multiplexed TMT proteomics (Figure S4). Similar to the plasma, many Ig isotypes were increased in old kidneys, including IgM, IgA, and IgGs (Figure 2A,B, Figure S6B, and Table S2). Other proteins that were particularly increased included Htra1 (serine protease), Umod (uromodulin), Mzb1 (marginal zone B- and B1-cell-specific protein), Mfge8 (lactadherin), Scrn1 (secernin-1), Ahsg (alpha-2-HS-glycoprotein), Bgn (biglycan), and Bpifa2 (BPI fold-containing family A member 2). Multiple proteins were also significantly decreased with age, including Mfap4 (microfibril-associated glycoprotein 4), Slc22a13 (solute carrier), Iba57 (putative transferase, mitochondrial), Cox11 (cytochrome c oxidase assembly protein, mitochondrial), Acox2 (peroxisomal acyl-coenzyme A oxidase 2), and Crot (peroxisomal carnitine O-octanoyltransferase).

We performed gene set enrichment analysis to evaluate which processes and pathways significantly changed with age. The biological processes most enriched with age were related to immune and defense response (e.g., adaptive immune response, lymphocyte-mediated immunity, phagocytosis, the regulation of B-cell activation, complement activation, and cytokine production) (Table S6a). The molecular functions that were most enriched included immunoglobulin receptor binding, signaling receptor binding, peptide antigen binding, and peptidase regulator activity (Figure 2C, Table S6b). For cellular component analysis, the extracellular region, extracellular matrix, collagen-containing extracellular matrix, immunoglobulin complex, and MHC protein complex were the most enriched in older mice, with the majority of differentially expressed proteins in these categories increasing (Table S6c). Proteins associated with the peroxisome were also enriched, with the majority showing a decrease with an increase in age. Analysis of ECM and ECM-associated proteins using the Matrisome database further highlighted that the majority of ECM proteins increased with increasing age (Figure S7B).

Similar to the plasma, we verified the findings in the kidney by profiling a separate cohort of 18 mice (9 young—3 months, 9 old—21 months) that showed similar increases in immunoglobulins with age (Figures S2 and S5B, Table S4). 

### 3.3. Immunoglobulins in Mouse Kidney Are Localized in Glomeruli

**Figure 3:**
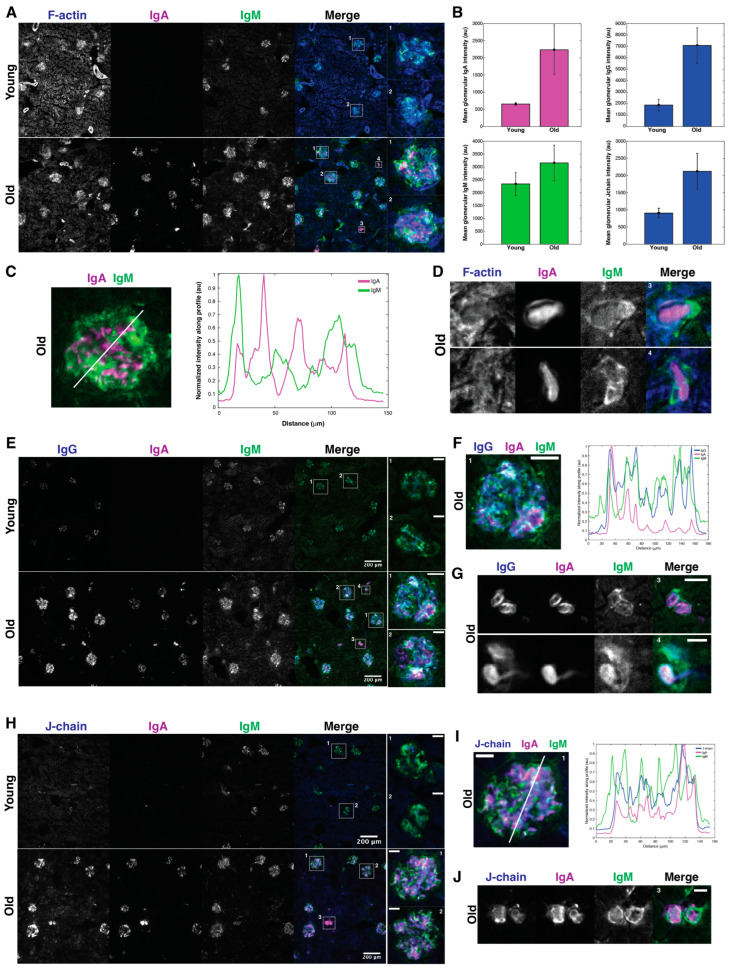
Glomerular and extra-glomerular IgG1, IgA, IgM, and J-chain deposits in old mouse kidneys. (**A**) IF image of IgA and IgM young (2 months) and old (18 months) mouse kidney sections. The boxed regions in the “Merge” panel highlight the glomeruli shown in the insets on the RHS or the extraglomerular regions shown on panel D. F-actin stained with fluorescently labeled phalloidin served as a guide to define the underlying kidney architecture, where glomeruli appear as distinct circular shapes with higher F-actin stain and cellular density. (**B**) Quantification of mean IgA, IgM, and IgG intensity for 35 young and 28 old glomeruli and mean J-chain intensity for 25 young and 18 old glomeruli. (**C**) The RHS panel shows the accumulation of IgA and IgM along the line profile as plotted over the glomerulus shown on the LHS panel. (**D**) IF image of IgA and IgM accumulation in the extraglomerular aggregates boxed and numbered in panel A. (**E**) IF image of IgG, IgA, and IgM on young (2 months) and old (18 months) mouse kidney sections. Boxed regions in the “merge” panel highlight the glomeruli shown in the insets on the RHS or the extraglomerular regions shown on panel G. (**F**) The RHS panel shows the accumulation of IgG, IgA, and IgM along the line profile, as plotted over the glomerulus shown on the LHS panel. (**G**) IF image of IgG, IgA, and IgM accumulation in the extraglomerular aggregates boxed and numbered in panel E. (**H**) IF image of J-chain, IgA, and IgM in young (2 months) and old (18 months) mouse kidney sections. The boxed regions in the “merge” panel highlight the glomeruli shown in the insets on the RHS or the extraglomerular region shown in panel J. (**I**) The RHS panel shows the accumulation of J-chain, IgA, and IgM along the line profile as plotted over the glomerulus shown on the LHS panel. (**J**) IF image of J-chain, IgA, and IgM accumulation in the extraglomerular aggregate boxed and numbered in panel H. Images are representative of all the data of kidney sections of *n* = 3 mice. All images were taken at 10× magnification. Scale bars as indicated.

To validate the increase in Igs in the kidneys with age and determine where the Igs localize in the kidney, we performed immunofluorescence (IF) microscopy. We confirmed that Igs increased with age in mouse kidneys and observed that they were mostly localized in the glomeruli according to F-actin staining (Figure 3 and Figure S11). The intensity was much greater in the glomeruli of the old mice than in the young mice (Figure 3A,B,E,H). Colocalization analysis revealed that the Ig isotypes were not all colocalized (Figure 3C,D,F,G,I,J). According to different combinations of IF staining, it appears that IgA is mostly colocalized with J-chain in the glomeruli (Figure S9), whilst IgM colocalizes well with IgG in the glomeruli (Figure S10), but there does not appear to be much overlap between IgA and IgM.

In addition to the Ig staining in the glomeruli, there appear to be some smaller areas that are also highly stained (Figure 3). We believe that these may be indicative of immune cell infiltrates, which have previously been reported to occur with age in multiple mouse organs [8], but additional IF staining for B and T cell markers is needed for confirmation.

### 3.4. Spatial Proteome Profiling Using Laser Capture Microdissection

**Figure 4:**
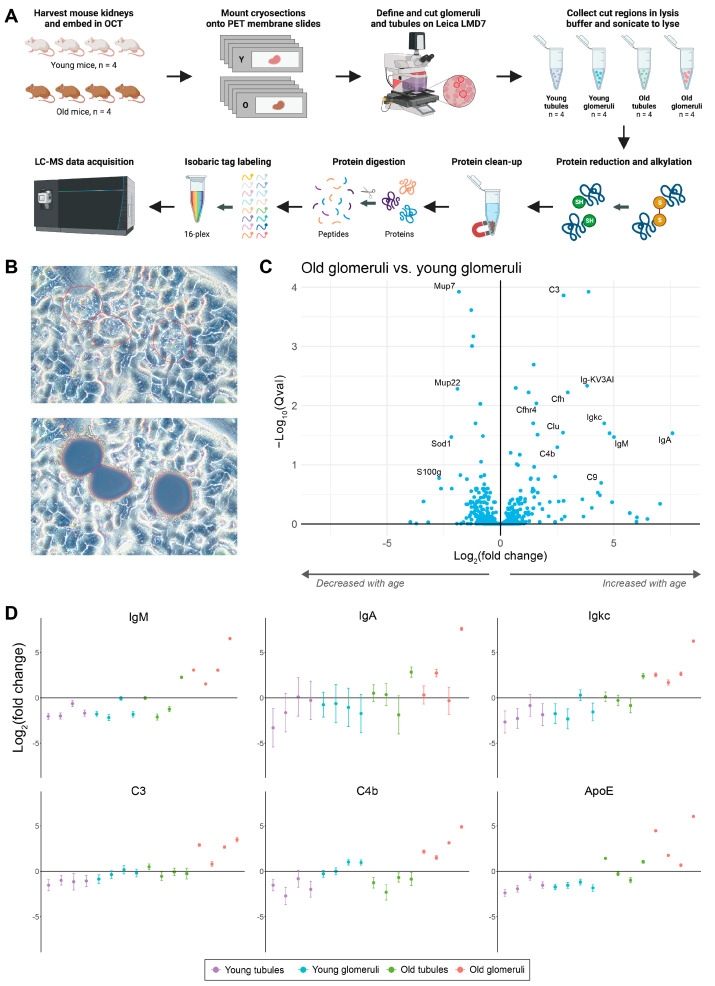
LCM analysis of aging mouse kidney glomeruli. (**A**) Experimental design for the comparison of tubules and glomeruli in young vs. old mice using laser capture microdissection coupled mass spectrometry. (**B**) Phase contrast microscopy images from Leica LMD7 of old kidney glomeruli at 20× before and after glomeruli were cut and collected. (**C**) Volcano plot showing changes between old glomeruli vs. young glomeruli for 1532 identified proteins (122 proteins with absolute fold change >2, 14 proteins with *q*-value < 0.01). (**D**) Scatter plots of differentially expressed proteins that are significantly increased in old glomeruli. (**A**) was created with Biorender.com (accessed on 30 January 2024).

To more comprehensively analyze the changes in different tissue structures and validate the IF results, we combined LCM with LC–MS proteomics to compare the changes in glomeruli and tubules in young and old mouse kidneys (Figure 4A).

Glomeruli and tubules were isolated from four young (2 months) and four old (18 months) mouse kidneys (Figure 4B). Comparing young and old glomeruli (Figure 4C) showed that various Igs, including IgM, IgA, and Igkc, were increased in the old glomeruli (Figure 4D). In addition, complement proteins C3 and C4b were markedly increased in the old glomeruli. Other complement components, including Cfh, Cfhr4, C9, and Clu, were also increased in old glomeruli. Comparisons between young and old tubules, young glomeruli and young tubules, and old glomeruli and old tubules also resulted in many differentially expressed proteins (Figure S3A–C). The comparison of glomeruli and tubules, without age as a factor, showed several differentially expressed proteins between the different kidney components (Figure S3D).

A global comparison was performed across the LCM glomeruli, whole plasma, and whole kidney LC–MS datasets to examine common significant protein changes with age (absolute fold change of >2, *q*-value of <0.05) (Figure S8). Igs, specifically IgM and Igkc, were the only proteins that were significantly increased with age across all three datasets. IgA was significantly increased in both old glomeruli and old kidneys, along with ApoE and Clu. Mup7 was the only protein that significantly decreased with age across all three datasets, while Mup8 significantly decreased in both the plasma and kidneys.

## 4. Discussion

### 4.1. Changes in High-Abundance Plasma Proteins with Age

We observed many significant differences in multiple high-abundance plasma proteins with age, making the case for the importance of analyzing neat, non-depleted plasma where these proteins are otherwise discarded. In particular, we observed large increases in Igs in the plasma with age as well as changes in complement proteins. 

It is not known how relevant the findings in mice are with respect to aging humans. While humans and mice share fundamental aspects of their immune system, there are notable differences in their Igs. There have been no large, neat plasma LC–MS profiling studies on aging human plasma published to date, but there have been studies using turbidimetric immunoassay-based methods. In the largest study of its kind, 8768 serum samples were profiled for IgG, IgA, and IgM [2]. The authors observed that IgA increased with higher age. IgG levels dipped around age 65 but then increased with older age, while IgM did not appear to change significantly.

It is important to point out the discrepancies that we observed between the changes in mouse plasma and human plasma with age. Increases in IgA with age appear to be fairly consistent between mouse and human plasma, with both increasing with age. However, the increase in IgM that we observed in mice does not seem to be consistent with the human plasma aging data, in which it is either observed to not change or decrease. The reason for this inconsistency between mice and humans is not known and needs to be explored further. The literature around IgG changes with age in humans is contradictory and is likely a result of differences in the exact isotypes (e.g., IgG1/2/3/4) that are quantified in the assays as well as the exact age range that is studied. A limitation of this study is that the mice profiled were from a single strain (C57/B6), and the observed changes may not be consistent across other mouse strains. Further experiments are needed on larger cohorts of mice and mice from different strains to confirm these findings. Similarly, large, unbiased proteome profiling of human plasma samples that use techniques that allow the profiling of abundant proteins including immunoglobulins are key to determining whether the findings in mice are consistent with those for plasma from human aging cohorts. 

In addition to high-abundance Ig proteins, many complement proteins were dysregulated with age. In particular, members of the membrane attack complex (C6, C8a, C8b C8g, and C9) along with Cfd (factor D) were significantly lower in the plasma in old age. Cfd is involved in the alternative complement pathway. The complement system is critical for innate immunity for the detection of foreign pathogens. A major part of the complement system is the membrane attack complex (MAC) which can be activated from any of the three complement pathways (classical, alternative, and lectin). The MAC comprises multiple complement proteins, including C5b, C6, C7, C8, and C9, and it is known to be present in both a soluble form in the plasma (sMAC) as well as in the more studied membrane form. Clusterin and vitronectin in the plasma prevent the membrane insertion of sMAC and inhibit cytolysis.

### 4.2. Changes in Kidney Proteins with Age

Optimal kidney function is critical for healthy aging, and biomarkers of mortality are often associated with impaired kidney function [18,19,20]. Premature aging is associated with impaired kidney filtration and kidney disease.

The proteins and pathways most increased in the aged kidney were related to inflammation, particularly the defense response. From the LC–MS data, Igs were shown to increase with age in the kidney. The Igs in the kidney could be due to increased deposition or the infiltration of B cells, and the specific localization of the increases in Igs in the kidney (e.g., tubules or glomeruli and immune cell infiltrates) was not known. Spatial proteomic techniques can help to gain an understanding of the localization of proteins in tissue structures. One of the most common spatial protein profiling techniques is immunofluorescence staining of proteins of interest from tissue slices using microscopy. However, this technique is limited to what antibodies are available for the proteins of interest. An alternative technique is laser capture microdissection (LCM), which enables unbiased proteome profiling of specific areas that can be dissected out under a microscope. We decided to perform spatial proteome profiling using a number of techniques to better understand the source and consequences of the increases in Igs with age. We performed both IF imaging and LCM proteomics to determine the localization of the Igs in the kidneys. Using IF imaging, we found that the Igs were localized predominantly in the glomeruli and were much higher in the older mice. In addition, there appeared to be small deposits in the older mice, which we believe are due to infiltrating immune cells (tertiary lymphoid organs). To determine whether the immunoglobulins were deposited in the same cells, we performed a colocalization analysis. IgM and IgG appeared to be mostly colocalized, whereas IgA was distributed separately in the glomeruli but did colocalize with J-chain. The LC–MS analysis of microdissected kidney tissue further validated that Igs were increased specifically in the glomeruli of old mice.

We hypothesized that the increased abundance of Igs with age observed in the kidney glomeruli is a result of the increased levels of Igs in the plasma leading to increased deposition in the kidney with age. However, the mechanisms behind this increased deposition in the kidney are not known. It could be a result of complement binding Igs, non-specific or charge-charge interactions, or increases in Ig receptors expressed in the kidney or glomeruli. Interestingly, Ig receptors including Fcgr4, Fcer1g, Fcgr2b, and Pigr were also observed to be increased in the kidney with age and may well play a role in the increased Ig deposition that is observed. However, since this is bulk profiling and not single-cell analysis, it is not clear whether there is an increase in the number of cells expressing those receptors or whether there is an increase in the expression of the receptors with the number of cells remaining constant.

### 4.3. Immunoglobulins

The source of the large increase in Igs with age that we observed in the plasma is not known. There are two main possible explanations: increased production or decreased clearance/turnover. It is not known which of these mechanisms is responsible, and it may well be a mix of the two and may differ depending on the specific Ig isotypes. 

It may be due to an increased production of Igs. B cells and plasma cells are responsible for producing Igs and may be the cause of the observed increase in Ig levels. There may be an increase in the total number of B/plasma cells with age, there may be increases in specific B cells with age (e.g., plasma cells), or there may be a change in the activation status of B cells with age. The majority of serum Igs are derived from B cells in the bone marrow, but to date, there has not been evidence of significantly increased numbers of B cells in the bone marrow with age [21,22]. Another possibility is that B cells outside of the bone marrow may play a role in the increase with age. For example, one possible explanation for the increased Ig levels in the plasma may be the increased levels of infiltrating B cells into organs. It is known that B cells infiltrate the kidney and other organs with age [19]. There are three main subsets of B cells: MZ, follicular (FO), and B-1 B cells. MZ B cells are primarily localized in secondary lymphoid organs, mainly in the marginal zone of the spleen, from which they derive their name. FO B cells are primarily localized in secondary lymphoid organs in B cell follicles. B-1 B cells are primarily localized in peritoneal and pleural cavities. MZ B cells are known to express poly-reactive BCRs and antibodies that are typically low-affinity. FO B cells typically express monoreactive BCRs that produce high-affinity antibodies. 

Interestingly, Mzb1 was significantly increased in old human kidneys. Mzb1 is expressed on plasma cells of the marginal zone (MZ) B cell subset, and Mzb1 may indicate an increased infiltration of plasma cells into the kidney with age or an increased activation state of B cells. Mzb1 is an ER-associated protein that is known to regulate B-cell receptor-driven calcium responses and regulates the assembly and secretion of IgM. Marginal zone (MZ) B cells, where Mzb1 is expressed, are predominantly found in the spleen, lymph nodes, and blood but there is evidence for them also being found in other organs, such as the kidney. MZ B cells are innate-like B cells, and their housekeeping function is the clearance of apoptotic cell debris as well as defense against blood-borne pathogens. There have been some reports that MZ B cells have been associated with autoimmunity and have previously been observed to be increased in peripheral blood B cells from patients with SLE [23], type 1 diabetes, rheumatoid arthritis, and Sjogren’s syndrome [24]. In autoimmune diseases, the overproduction of Igs from autoreactive B cells is often considered the mechanism for the observed increase in serum Igs, and a similar mechanism may be at play in aging as well.

Alternatively, other possible explanations could be that the lifespan of the Igs changes with age. For example, there could be impaired clearance of Igs/immune complexes with age. Impaired clearance may result in the accumulation of Igs in tissues and could be an explanation for the increased deposition of Igs in the kidneys.

The clearance mechanisms for all the different immunoglobulin isotypes are not precisely known, and it could be that some of these mechanisms become impaired with age. The clearance of the Igs may change depending on different proteoforms or changes in the structure or binding partners of the Igs. For example, Ig glycosylation has been reported to change with age [25], and it is known that differences in glycosylation influence their half-life and may play a role in changes in clearance with age. Alternatively, changes in the expression of Ig receptors on the cells responsible for the removal of immunoglobulins may change with age.

The impact of the high Ig levels observed with age is not known. In humans, low Ig levels (e.g., Ig deficiencies) are known to be associated with an increased risk of infection. High plasma Ig levels have previously been associated with highly inflammatory diseases, such as autoimmune diseases (e.g., rheumatoid arthritis and systemic lupus erythematosus), kidney disease, liver disease, allergies, chronic infections, or certain cancers, such as B-cell lymphomas. Igs are known to be deposited in multiple tissues in the body, with the kidney reported to be particularly susceptible to damage by immunoglobulin deposits.

With age, it is known that there is an increased vulnerability to infection and that older adults are less able to mount an effective response to vaccination. This would appear to be contradictory to the observed increase in Igs that we observed in our data. However, it is important to point out that it is the ability to generate high-affinity antibodies against the infection that is critical for defense against infections rather than just the quantity of Igs in the plasma. With age, the ability to generate high-affinity antibodies against antigens is known to be impaired and is likely a result of B cell/T cell dysfunction and senescence with age [26].

### 4.4. Complement

The complement system comprises a complex network of proteins that contribute to immune defense, inflammation, and tissue repair. C3, one of the proteins observed to be most increased in old glomeruli, is a key component of this system and serves as a central hub by initiating both the classical and alternative complement pathways. In its activated form, C3 facilitates the opsonization of particles and pathogens, which enhances the recognition and phagocytosis of these particles by macrophages within the kidney, preventing the spread of infection and maintaining tissue integrity.

Interestingly, in the bulk proteomics data, there were no major changes in the levels of C3 or C4b. However, in the glomeruli, C3 and C4b were among the top proteins to be significantly increased with age, showcasing the power of spatial over bulk proteomics to identify structure-specific changes with age. However, in the bulk kidney tissue, there were major changes in many complement-related proteins, including significant increases in C1qc, C1qb, C1qa, C1ra, C5, C6, C9, Clu, Vtn, Cfd, and Cfh, suggesting major complement activation/dysfunction with age.

Various glomerular diseases are associated with the deposition of C3 in the glomeruli. For example, membranoproliferative glomerulonephritis (MPGN) is characterized by the accumulation of C3 in the glomerular basement membrane [27]. This deposition can result from the dysregulation of the complement system or the formation of immune complexes within the glomerulus. Immune complexes are formed when antigens (foreign substances or self-antigens) interact with antibodies. C3 can be deposited in the kidney when these immune complexes, consisting of antigens and antibodies, are filtered through the glomerulus. It is known that in autoimmune diseases (e.g., lupus and rheumatoid arthritis), deposition of C3 in the kidney can occur. Deposition of C3 in kidneys is often associated with complement activation, inflammation, and tissue damage. This may suggest that there is activation/dysregulation of the complement system with age, resulting in large amounts of C3 deposition in the kidney which could be a result of chronic inflammation.

## 5. Conclusions

Through proteome profiling of neat (undepleted) young and old mouse plasma using LC–MS, we discovered that Igs were the proteins most increased with age in old plasma. This observation appears to have been overlooked in the aging proteome profiling literature as most of the studies performed to date have used either targeted assays (e.g., Olink/SomaLogic assays) or have used depletion approaches that remove the high-abundant proteins. We also observed major changes in complement proteins with age. We propose that strategies utilizing neat plasma LCMS are valuable as insights into innate and adaptive immune responses may otherwise be lost if the proteins of high abundance are ignored. Profiling of Igs will likely be useful as biomarkers of inflammation and inflammaging. Larger cohort studies profiling Igs in both mice and humans are needed to determine the utility of Igs as possible readouts of biological age.

Following up on the possible consequences of high Ig levels in plasma, we profiled the kidney, the organ that is responsible for filtering plasma proteins and known to be sensitive to protein deposition from the plasma. Increases in Igs were observed in old kidneys and were localized predominantly in the glomeruli. Further analysis of the glomeruli using LCM confirmed this observation and showed large increases in complement-associated proteins with age, including C3, Cfh, Clu, and C9. In bulk kidney profiling, C3 was not observed to be significantly increased, whilst it was when performing LCM on just the glomeruli, highlighting the power of spatial proteomics over bulk proteome profiling.

Increased Ig levels in plasma likely lead to increased deposition of Igs, immune complexes, and complement in the kidney with age. This deposition is primarily localized to the glomeruli and likely plays a role in the reduced filtering capacity of the kidney with age. Therapeutic strategies to reduce deposition and enhance the removal of deposited proteins would likely improve kidney filtering capacity and longevity.

## 6. Limitations

The number of mice profiled in this study was relatively small. However, efforts were made to profile mice from separate studies to determine if similar changes were observed. The first set used 10 mice (5 young, 5 old), and the second set used 18 mice (9 young, 9 old). In this study, only unmodified proteins were measured and not proteoforms.

## Figures and Tables

**Table 1:** Table of antibodies used for immunofluorescence.

**Primary**	**Catalog Number**
Goat anti-mouse IgA alpha chain (DyLight-594)	Abcam (Cambridge, UK), ab97013
Rat anti-mouse IgM mu chain (SB73a)—Abcam ab99600	Abcam, ab99600
Goat anti-mouse IgG H&L (Alexa Fluor^®^ 488)	Abcam, ab150113
Rabbit anti-human J-Chain	ThermoFisher, MA5-16419
**Secondary**	**Catalog Number**
Donkey anti-Goat IgG (H + L) Cross-Adsorbed Secondary Antibody, Alexa Fluor 568	ThermoFisher, A-11057
Donkey anti-Rat IgG (H + L) Highly Cross-Adsorbed Secondary Antibody, Alexa Fluor Plus 488	ThermoFisher, A-48269

## Data Availability

The mass spectrometry proteomics data have been deposited to the ProteomeXchange Consortium via the PRIDE [1] partner repository with the dataset identifiers PXD051031 (Kidney) and PXD051031 (plasma).

## Supplementary Materials

The following supporting information can be downloaded at: https://www.mdpi.com/article/10.3390/proteomes12020016/s1, Figure S1: Plasma proteome profiling of 9 young and 9 old mice shows increase in immunoglobulins with age; Figure S2: Kidney proteome profiling of 9 young and 9 old mice shows increase in immunoglobulins with age; Figure S3: Comparison of differentially expressed proteins in tubules and glomeruli from young and old mice; Figure S4: Experimental design for multiplexed plasma proteome profiling of 5 young and 5 old mice; Figure S5: Number of quantified proteins in plasma and kidney LC-MS datasets; Figure S6: Immunoglobulin (Ig) proteins identified in mouse plasma and kidney; Figure S7: Extracellular matrix (ECM) proteins identified in mouse plasma and kidney; Figure S8: Comparison of significantly changing proteins with age across whole plasma, whole kidney and laser capture microdissected (LCM) glomeruli; Figure S9: Glomerular IgA and J-chain enrichment in old mouse kidneys; Figure S10: Glomerular IgG1 and IgM enrichment in old mouse kidneys; Figure S11: F-actin and DAPI reveal underlying kidney architecture; Table S1: Fold changes for old vs. young plasma (10-plex); Table S2: Fold changes for old vs. young kidney (10-plex); Table S3: Fold changes for old vs. young plasma (18-plex); Table S4: Fold changes for old vs. young kidney (18-plex); Table S5a: Gene Ontology Biological Processes for old vs. young plasma; Table S5b: Gene Ontology Molecular Functions for old vs. young plasma; Table S5c: Gene Ontology Cellular Components for old vs. young plasma; Table S6a: Gene Ontology Biological Processes for old vs. young kidney; Table S6b: Gene Ontology Molecular Functions for old vs. young kidney; Table S6c: Gene Ontology Cellular Components for old vs. young kidney.
