# Supplementary material for: Plasma and Kidney Proteome Profiling Combined with Laser Capture Microdissection Reveal Large Increases in Immunoglobulins with Age
Source: Proteomes. 2024 Jun 3;12(2):16. doi: 10.3390/proteomes12020016 (PMC11207650; doi:10.3390/proteomes12020016)
Supplement: Supplementary file 1 [file proteomes-12-00016-s001.zip › LCM_paper_figures_SUPP_FINAL_02APR2024.pdf]

Supplementary Information for

Plasma and kidney proteome profiling combined with laser capture microdissection reveal large increases in immunoglobulins with age

Leanne J.G. Chan, Niclas Olsson, Magdalena Preciado López, Kayley Hake, Haruna Tomono, Matthew A. Veras and Fiona E. McAllister *

Figures S1a, S3a, S4 were created with Biorender.com

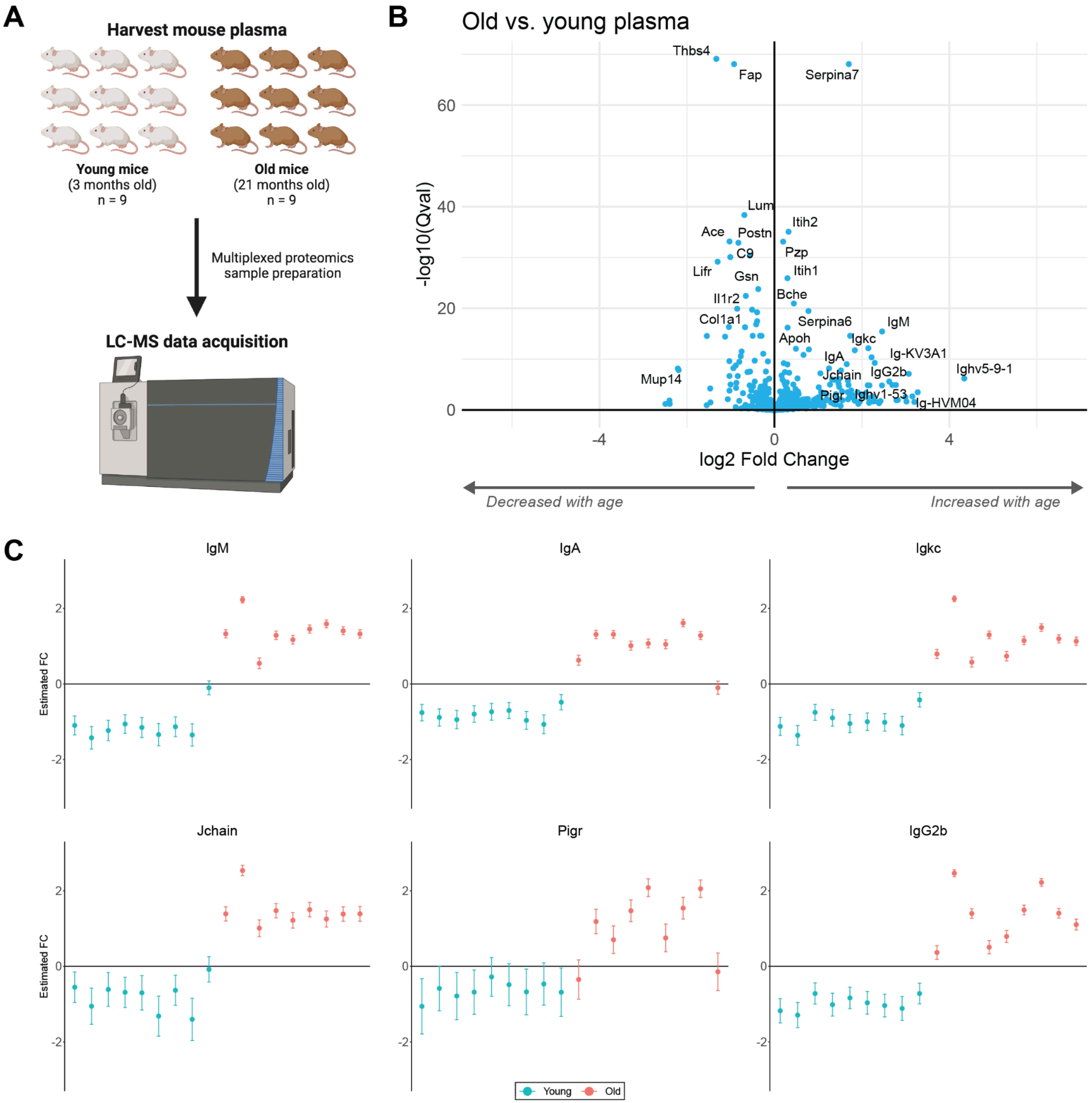

Figure S1. Plasma proteome profiling of 9 young and 9 old mice shows increase in immunoglobulins with age.
(A) Experimental design for multiplexed plasma proteome profiling of 9 young and 9 old mice. (B) Volcano plot showing changes between old versus young plasma for 692 identified proteins (100 proteins with absolute fold change > 2, 188 proteins with q-value < 0.01). (C) Scatter plots of proteins increased in old plasma.

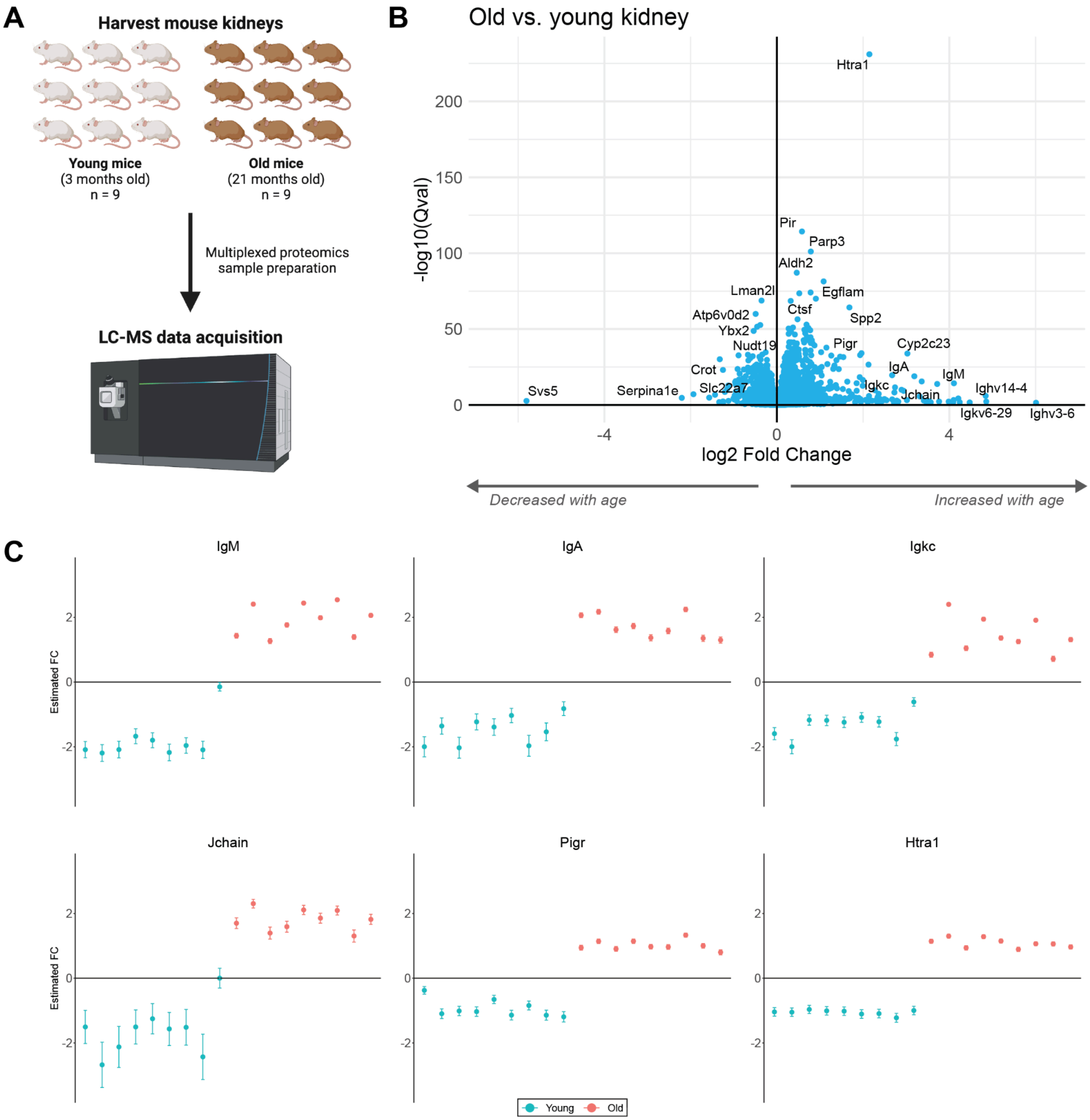

Figure S2. Kidney proteome profiling of 9 young and 9 old mice shows increase in immunoglobulins with age. (A) Experimental design for multiplexed kidney proteome profiling of 9 young and 9 old mice. (B) Volcano plot showing changes between old versus young kidneys for 9,841 identified proteins (163 proteins with absolute fold change > 2, 3,522 proteins with q-value < 0.01). (C) Scatter plots of proteins increased in old kidneys.

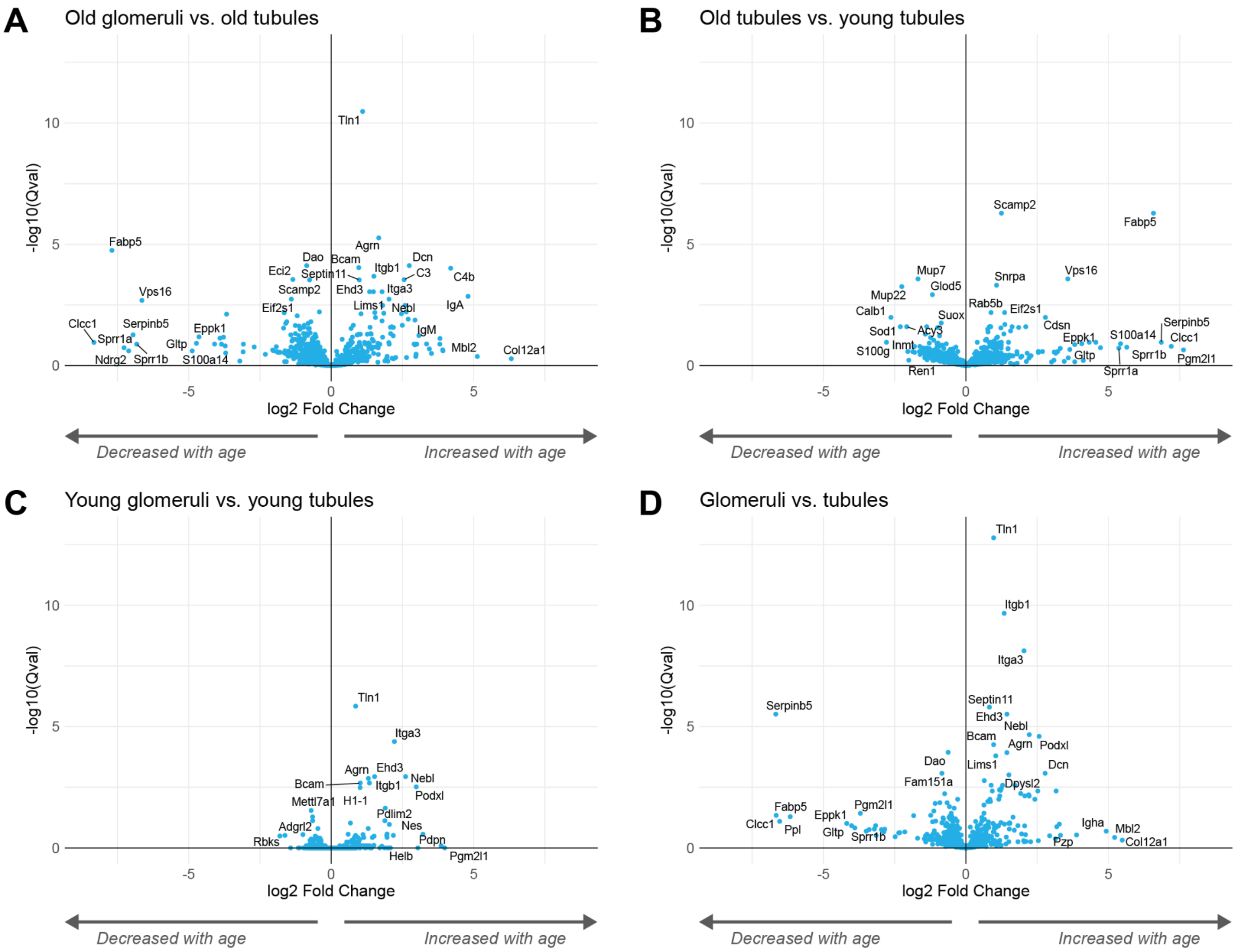

Figure S3. Comparison of differentially expressed proteins in tubules and glomeruli from young and old mice.

(A) Volcano plot showing changes between old glomeruli versus old tubules for 1,532 identified proteins (205 proteins with absolute fold change > 2, 30 proteins with q-value < 0.01). (B) Volcano plot showing changes between old tubules versus young tubules for 1,532 identified proteins (200 proteins with absolute fold change > 2, 9 proteins with q-value < 0.01). (C) Volcano plot showing changes between young glomeruli versus young tubules for 1,532 identified proteins (52 proteins with absolute fold change > 2, 9 proteins with q-value < 0.01). (D) Volcano plot showing changes between glomeruli versus tubules without age factor for 1,534 identified proteins (115 proteins with absolute fold change > 2, 35 proteins with q-value < 0.01).

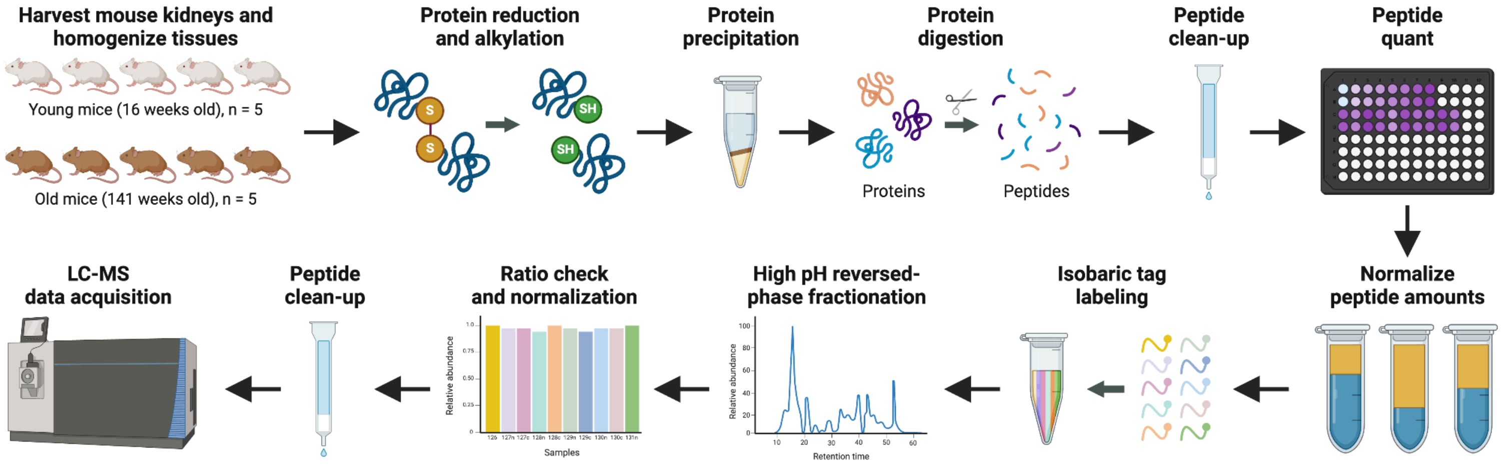

Figure S4. Experimental design for multiplexed plasma proteome profiling of 5 young and 5 old mice.

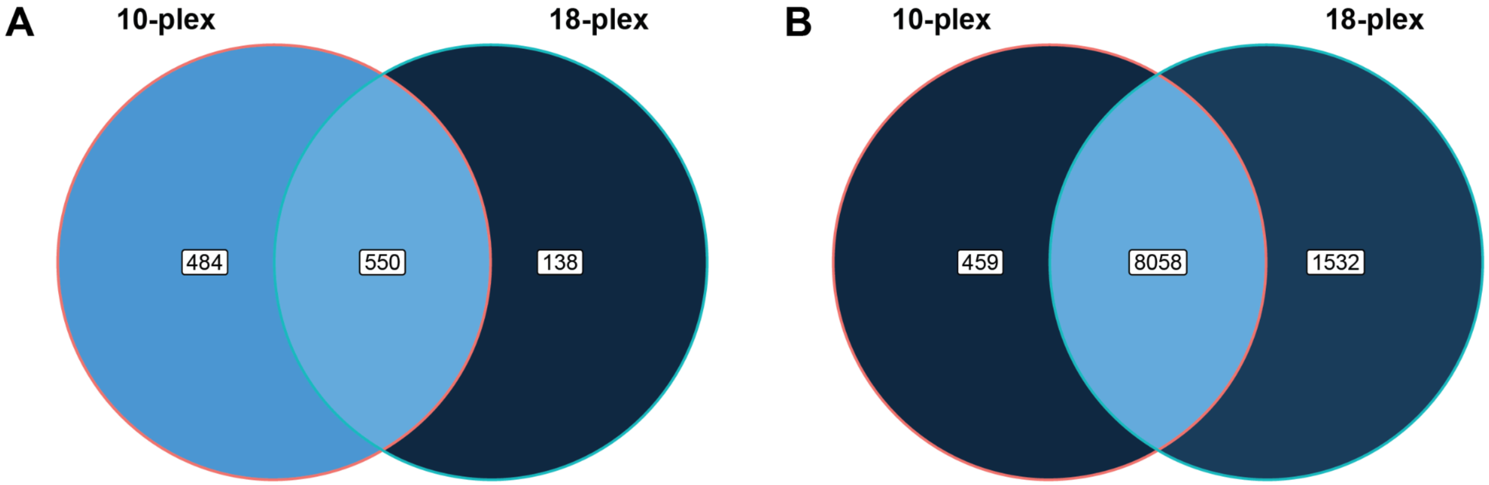

Figure S5. Number of quantified proteins in plasma and kidney LC-MS datasets.

(A) Venn diagram showing number of quantified proteins shared between 10-plex and 18-plex plasma profiling experiments. (B) Venn diagram showing number of quantified proteins shared between 10-plex and 18-plex kidney profiling experiments.

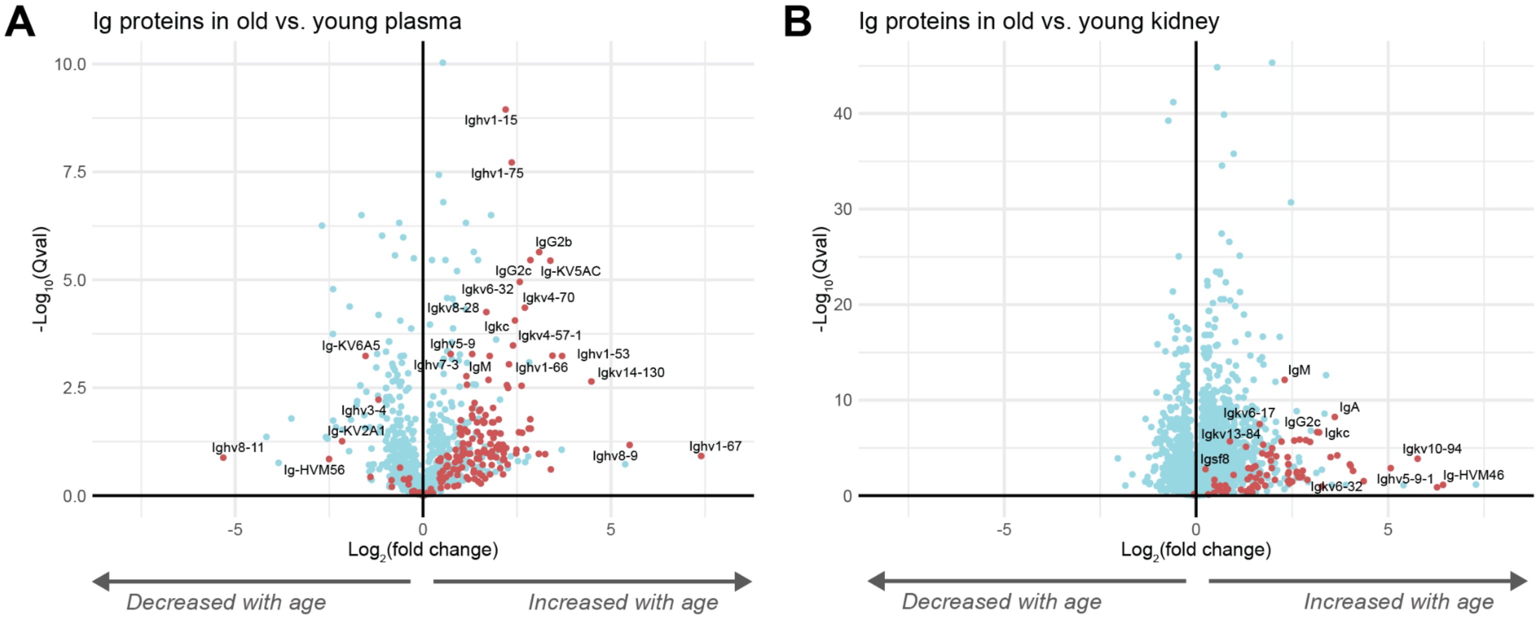

Figure S6. Immunoglobulin (Ig) proteins identified in mouse plasma and kidney.

(A) Volcano plot showing changes between young and old mouse plasma for 1,044 identified proteins. Immunoglobulin protein identifications are highlighted in red: 169 total proteins, 116 proteins with absolute fold change > 2, 28 proteins with q-value < 0.01. (B) Volcano plot showing changes between young and old mouse kidney for 8,684 identified proteins. Immunoglobulin protein identifications are highlighted in red: 86 total proteins, 68 proteins with absolute fold change > 2, 44 proteins with q-value < 0.01.

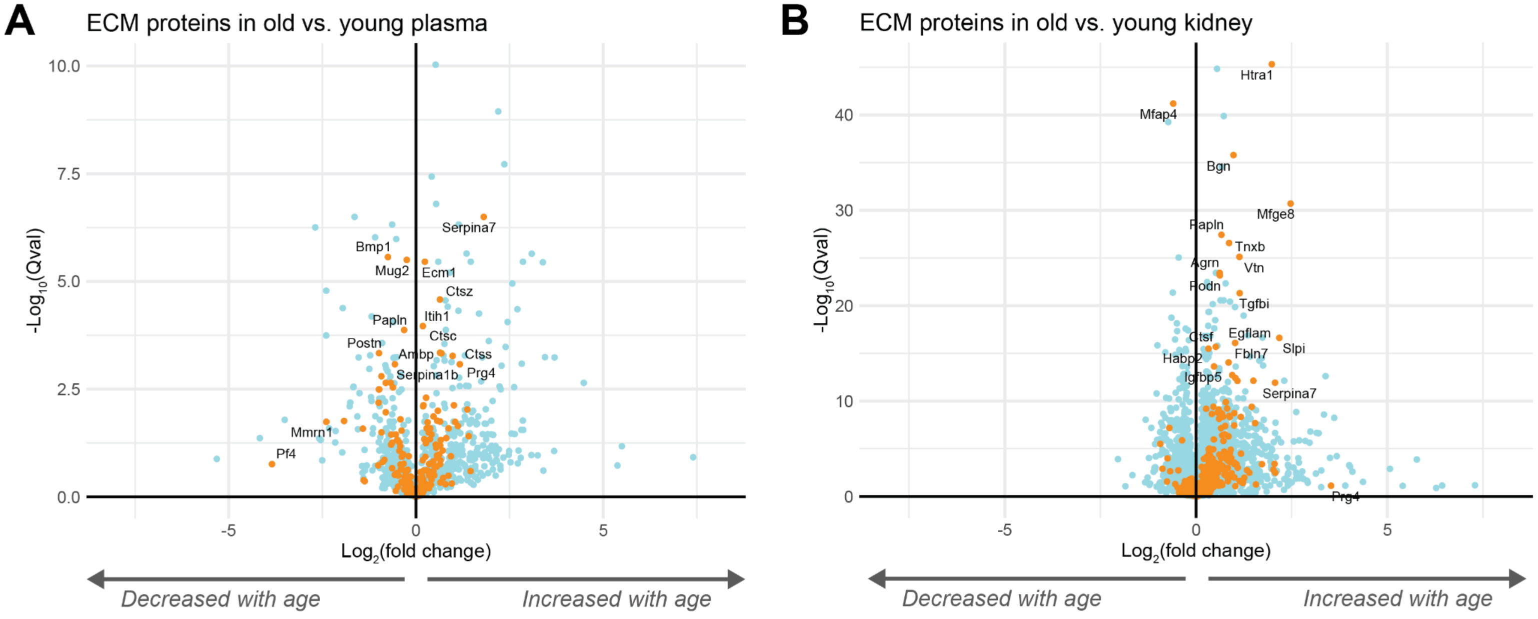

Figure S7. Extracellular matrix (ECM) proteins identified in mouse plasma and kidney.

(A) Volcano plot showing changes between young and old mouse plasma for 1,044 identified proteins. ECM protein identifications are highlighted in orange: 208 ECM proteins identified, 15 proteins with absolute fold change > 2, 25 proteins with q-value < 0.01. (B) Volcano plot showing changes between young and old mouse kidney for 8,684 identified proteins. ECM protein identifications are highlighted in orange: 315 ECM proteins identified, 34 proteins with absolute fold change > 2, 140 proteins with q-value < 0.01.

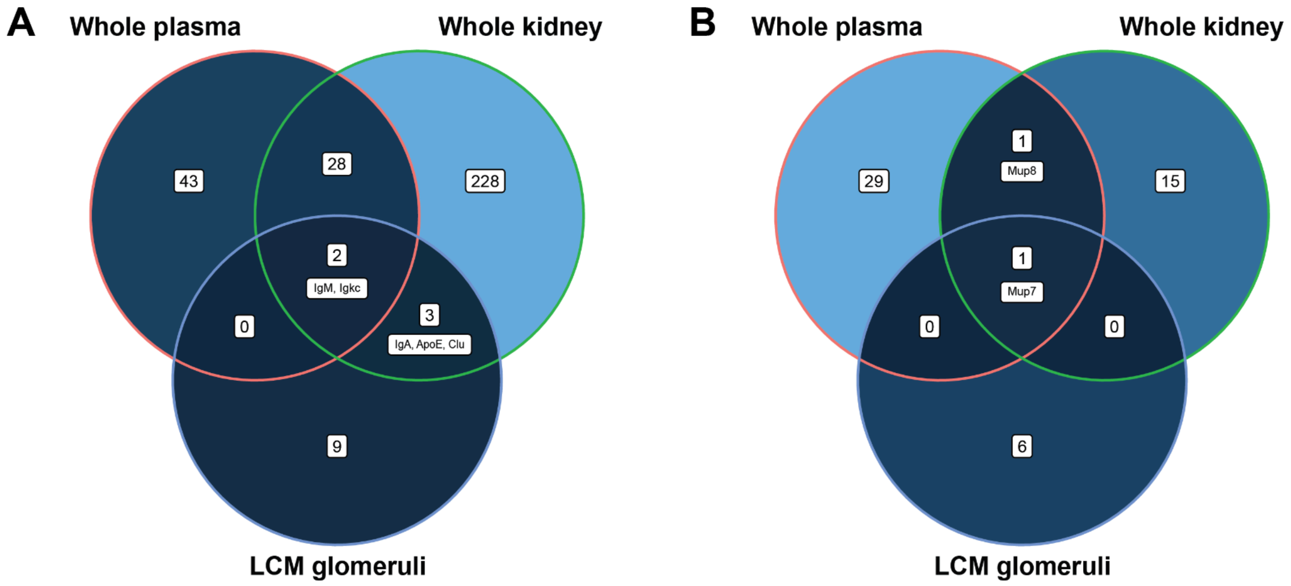

Figure S8. Comparison of significantly changing proteins with age across whole plasma, whole kidney and laser capture microdissected (LCM) glomeruli

(A) Venn diagram showing overlap of proteins significantly increased with age across 10-plex plasma, 10-plex kidney and LCM glomeruli LC-MS experiments (fold change > 2, q-value < 0.05). (B) Venn diagram showing overlap of proteins significantly decreased with age across 10-plex plasma, 10-plex kidney and LCM glomeruli LC-MS experiments (fold change < -2, q-value < 0.05).

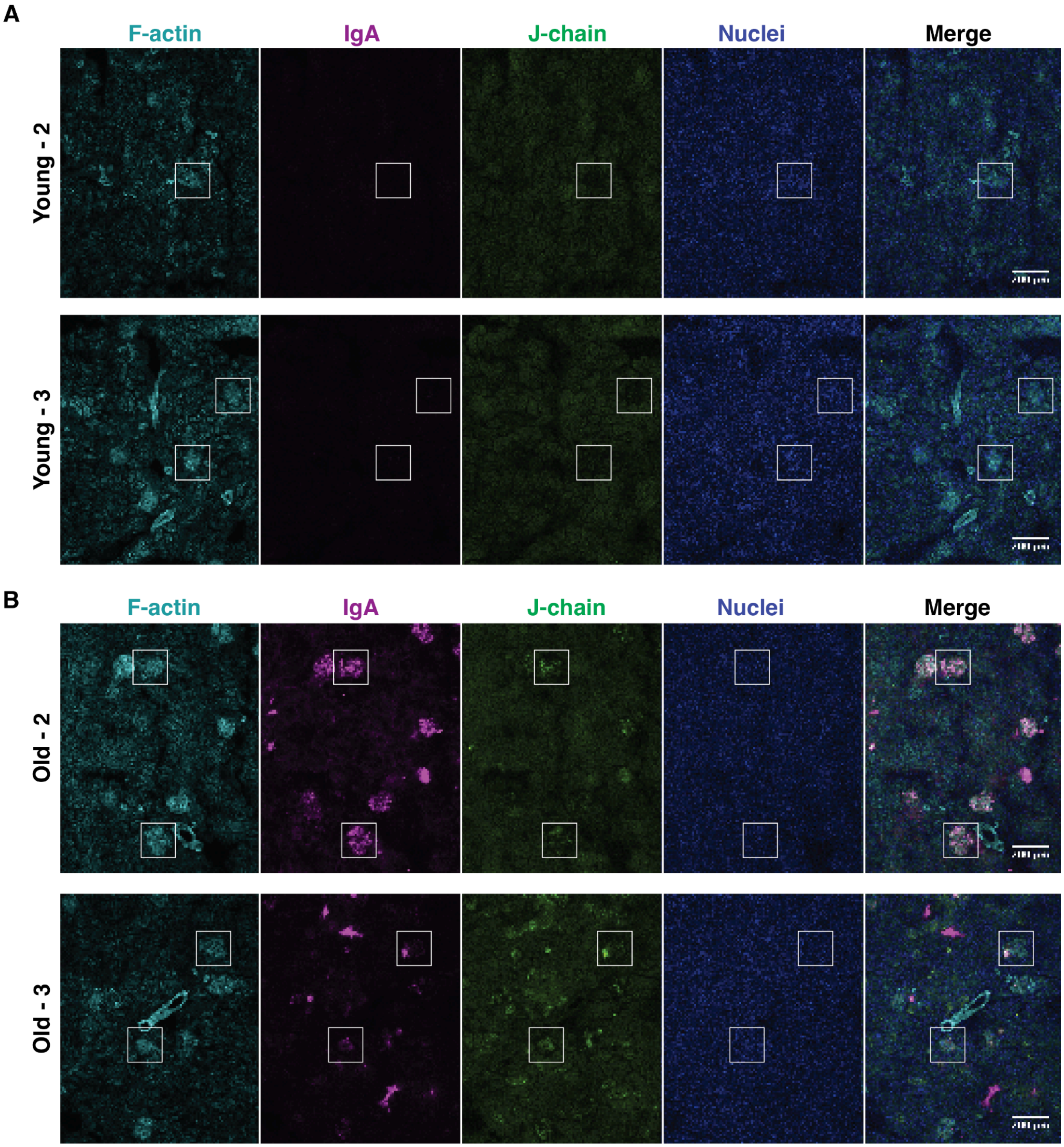

Figure S9. Glomerular IgA and J-chain enrichment in old mouse kidneys.

(A) Immunofluorescence images of IgA and J-chain localization in kidney sections of two young (2 months) mice. (B) Immunofluorescence images of IgA and J-chain localization in kidney sections of two old (18 months) mice. In all panels, umbered boxed regions indicate glomeruli across all channels and the merge panel. F-actin stained with fluorescently-labeled phalloidin and nuclei with DAPI served as guides to define the underlying kidney architecture, where glomeruli appear as distinct circular shapes with higher F-actin concentration and nuclear density. Images are representative of all of the data collected from kidney sections of n=3 mice. Images were taken at 20x magnification. Scale bars as indicated.

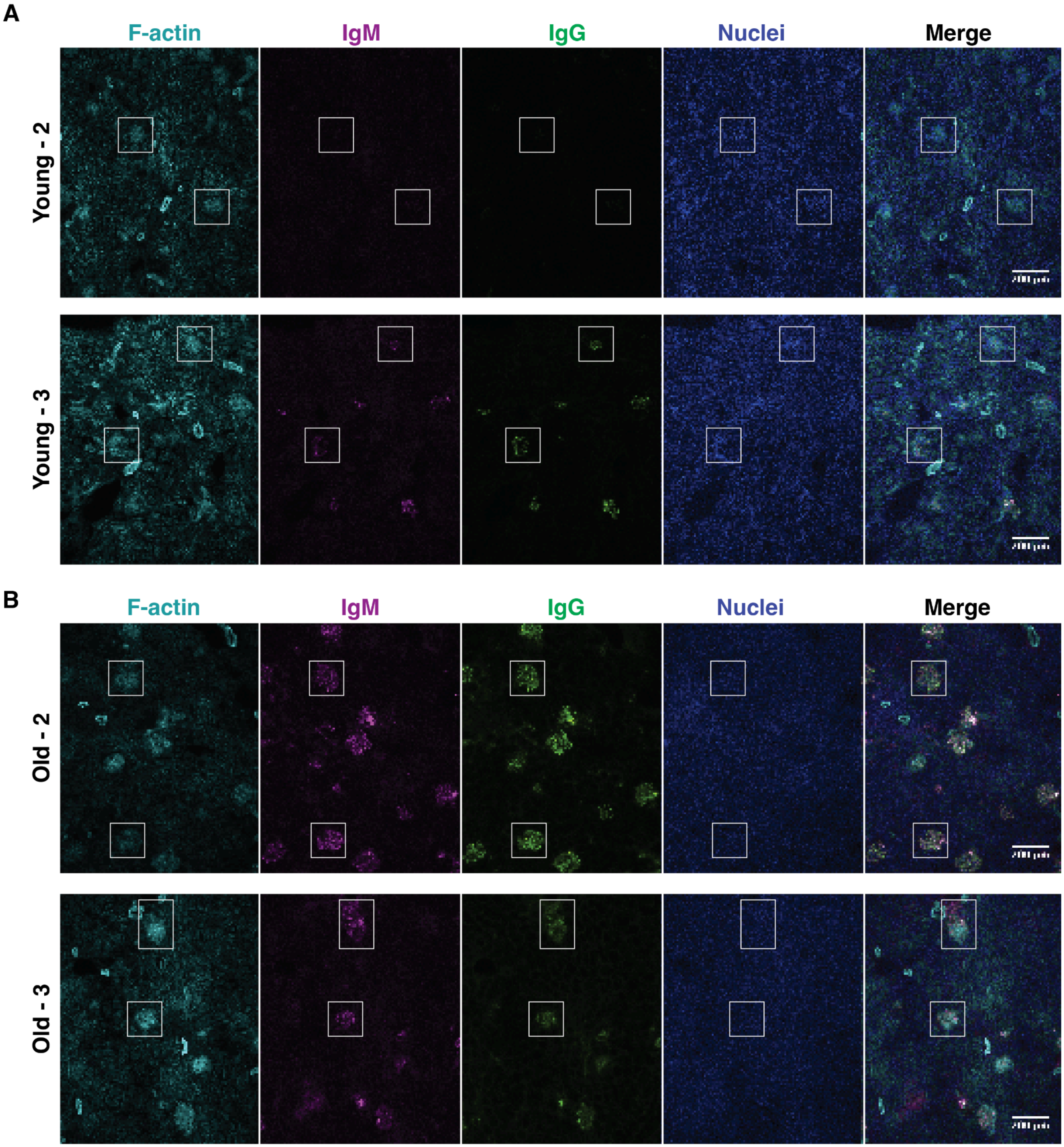

Figure S10. Glomerular IgG1 and IgM enrichment in old mouse kidneys.

(A) Immunofluorescence images of IgG1 and IgM localization in kidney sections of two young (2 months) mice. (B) Immunofluorescence images of IgG1 and IgM localization in kidney sections of two old (18 months) mice. In all panels, numbered boxed regions indicate glomeruli across all channels and the merge panel. F-actin stained with fluorescently labeled phalloidin, and nuclei with DAPI served as guides to define the underlying kidney architecture, where glomeruli appear as distinct circular shapes with higher F-actin concentration and nuclear density. Images are representative of all of the data collected from kidney sections of n=3 mice. Images were taken at 20x magnification. Scale bars as indicated.

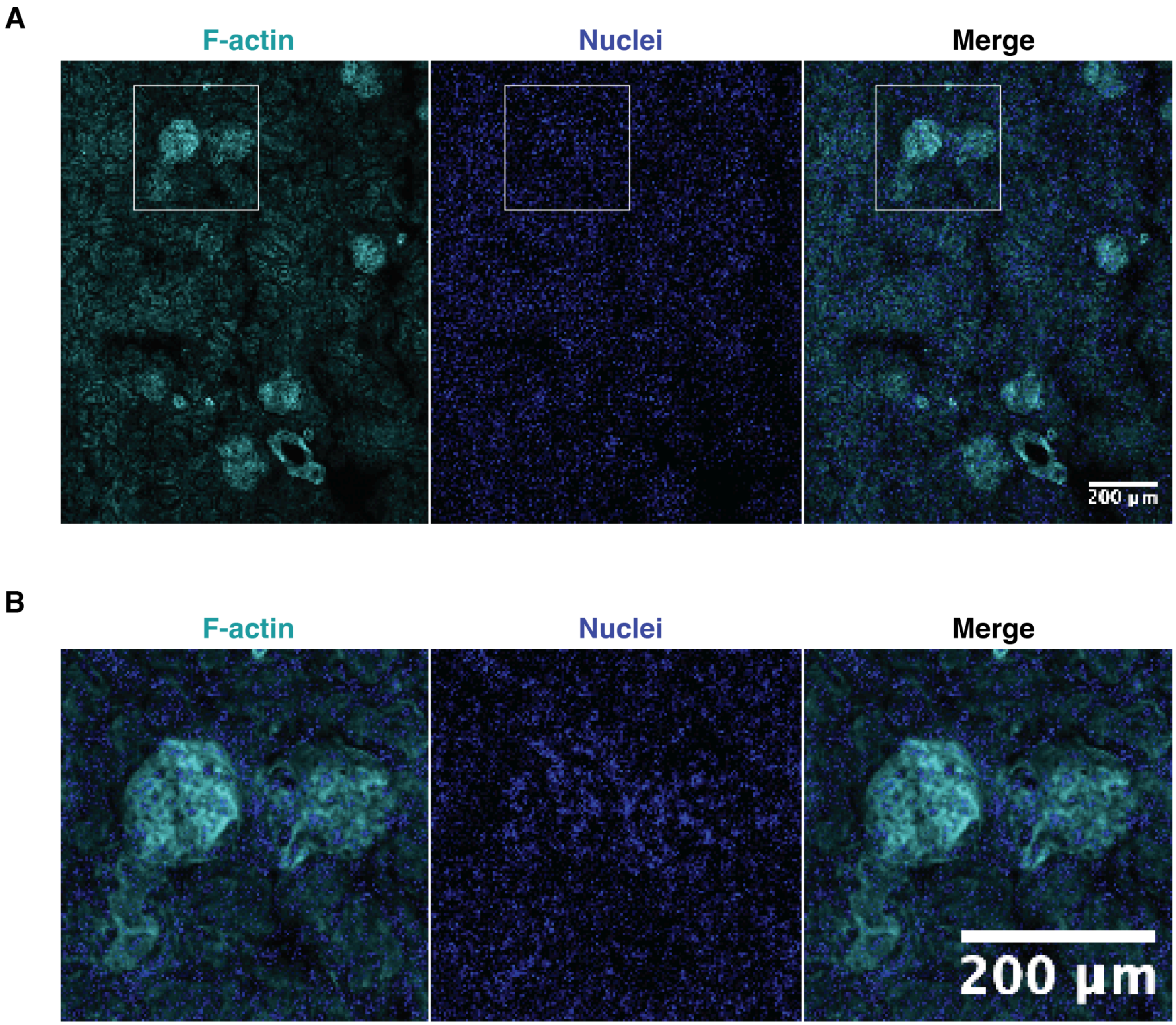

Figure S11. F-actin and DAPI reveal underlying kidney architecture.

(A) Representative immunofluorescence image of F-actin (stained with fluorescently labeled phalloidin) and nuclei (stained with DAPI) of kidney sections from a young (2 months) mouse. Glomeruli appear as distinct circular shapes with higher F-actin concentration and nuclear density than the surrounding regions. (B) Magnified image of the boxed region in panel (A) two glomeruli side by side. Images are representative of all of the data collected from kidney sections of n=3 mice. Images were taken at 20x magnification. Scale bars as indicated.
